# The composition of Australian *Plantago* seeds highlights their potential as nutritionally-rich functional food ingredients

**DOI:** 10.1038/s41598-021-92114-1

**Published:** 2021-06-16

**Authors:** James M. Cowley, Lisa A. O’Donovan, Rachel A. Burton

**Affiliations:** 1 Australian Research Council Centre of Excellence in Plant Cell Walls, School of Agriculture, Food and Wine, University of Adelaide, Waite Campus, Urrbrae, SA Australia; 2 Australian Research Council Centre of Excellence in Plant Energy Biology, School of Agriculture, Food and Wine, University of Adelaide, Waite Campus, Urrbrae, SA Australia

**Keywords:** Natural variation in plants, Cell wall, Nutrition

## Abstract

When wetted, *Plantago* seeds become covered with a polysaccharide-rich gel called mucilage that has value as a food additive and bulking dietary fibre. Industrially, the dry husk layer that becomes mucilage, called psyllium, is milled off *Plantago ovata* seeds*,* the only commercial-relevant *Plantago* species, while the residual inner seed tissues are either used for low value animal feed or discarded. We suggest that this practice is potentially wasting a highly nutritious resource and here describe the use of histological, physicochemical, and chromatographic analyses to compare whole seed composition/characteristics of *P. ovata* with 11 relatives already adapted to harsh Australian conditions that may represent novel commercial crop options. We show that substantial interspecific differences in mucilage yield and macromolecular properties are mainly a consequence of differences in heteroxylan and pectin composition and probably represent wide differences in hydrocolloid functionality that can be exploited in industry. We also show that non-mucilage producing inner seed tissues contain a substantial mannan-rich endosperm, high in fermentable sugars, protein, and fats. Whole seed *Plantago* flour, particularly from some species obtained from harsh Australian environments, may provide improved economic and health benefits compared to purified *P. ovata* psyllium husk, by retaining the functionality of the seed mucilage and providing additional essential nutrients.

## Introduction

Plants of the genus *Plantago* occur widely throughout the world. Traditionally, all parts of the plant are used^1–5^, but the seeds have particular importance as they produce a gel-like coating of mucilage upon wetting which has many folk food and medicinal uses. For example, seeds of Australian *Plantago* species were pounded into a flour by Aboriginal and Torres Strait Islander peoples and combined with water to make a porridge thickened by the sticky mucilage^6,7^. Early British settlers also noted the palatability of Australian native *Plantago* seeds, exploiting the jelly-like mucilage to prepare sweetened desserts similar to sago pudding^8^. More recently, *Plantago* seed mucilage has gained great industrial and medical significance. Commonly known as psyllium husk, the milled seed mucilage of *P. ovata* contains highly-hydrophilic heteroxylan and pectin polysaccharides^9,10^ that are used in food technology to texturally mimic fat^11^ and gluten^12,13^, and in health applications as a dietary fibre supplement to aid laxation^14^, treat hypercholesterolemia^15^, diabetes^16^, and irritable bowel syndrome^17^. While the clinical benefits of psyllium husk (generally attributed to the high viscosity of the heteroxylan) are not in doubt, pre-clinical and in vitro studies showing free radical scavenging^18–23^, immunomodulation^24–26^, and treatment of metabolic disorders^27–30^ by extracts of whole, unhusked *Plantago* seeds demonstrate that the non-husk/non-mucilage seed components may have further beneficial effects. However, current production of psyllium husk from *P. ovata* (the only commercially important *Plantago* species) mostly discards these non-husk/non-mucilage components which account for around 75% of the seed yield^10^. In India (the world’s largest psyllium producer) this equates to over 160,000 tonnes of non-husk material that was wasted from the 56,000 tonnes of psyllium husk produced in 2019/2020^31,32^ despite the material likely containing many beneficial nutrients. It is likely that a major factor contributing to the underutilisation of whole *Plantago* seeds is that remarkably little is known about whole seed composition, particularly from a nutritional perspective, and how the composition differs between species. Natural interspecific variation in the cosmopolitan *Plantago* genus has already been a valuable resource to study polysaccharide biosynthesis^33–35^ but is now increasing in interest as a source of novel ingredients with unique functional properties for industry^19,36–41^. The limited studies that are currently available have shown that whole seed flour (WSF) from *P. ovata* and *P. psyllium* are useful hydrocolloid replacements in baking^42,43^ with significant differences in product quality between the two species. These studies show the potential of WSF from different *Plantago* species to be a less wasteful, more nutritious alternative to psyllium husk for selectively manipulating food qualities based on interspecific differences in functionality. Therefore, we hypothesise that different *Plantago* species may yield correspondingly diverse functionalities and Australia may boast valuable untapped resources, with the added advantage of already being adapted to demanding climatic constraints that limit production of psyllium from *P. ovata*^10^.

Thus, the aim of this work was to compare the composition and morphology of seeds of commercial psyllium, *P. ovata*, to eleven related species naturalised or native to Australia to assess their nutritional and functional potential as novel food ingredients. The results demonstrate great diversity in mucilage properties and composition that likely represent exploitable differences in hydrocolloid functionality and have provided the first comprehensive overview of *Plantago* seed composition, showing them to be rich in beneficial sugars, fats and protein. We suggest that the use of WSF from diverse *Plantago* species studied here will simultaneously allow selective manipulation of quality and nutritional enrichment of food products.

## Materials and methods

### Plant growth for seed bulking

Seeds of twelve *Plantago* species were obtained from the sources listed in Supplementary Table 1, and where applicable, collection complied with relevant institutional, national, and international guidelines and legislation. Seeds were stratified dry for 48 h at − 20 °C prior to germination then imbibed in filtered (0.22 µm) sterilisation agent (50:50, 50% ethanol:4% bleach with 0.05% Triton X-100) for 1 min before replacing the sterilisation agent and incubating for another minute. This was repeated until the seeds had been imbibed in fresh sterilisation agent 5 times, after which the seeds were washed 5 times with filter-sterilised (0.22 µm) Milli-Q water. Seeds were spread onto pre-wetted autoclaved Whatman No. 1 paper in a sterile petri dish. Dishes were sealed, aluminium foil-wrapped and stratified for another 48 h at 4 °C. After stratification, plates were moved to a glasshouse with a day/night temperature of 23 °C/18 °C. Seeds were germinated for 10 days (3 days dark then 7 days exposed to the glasshouse day/night light cycle) then transferred to coco-peat soil mixture in tall citrus pots. Plants were grown to maturity from June to December (Adelaide, Australia) with no supplemental light. Once mature (capsules freely dehiscing), inflorescences were harvested, dried for 48 h at 37 °C, and then threshed to obtain seed. Where required, whole seeds were ground using a MM400 Mixer Mill (Retsch, Germany) and graded to 0.5 mm to produce whole seed flour (WSF).

### Phylogenetic analysis

Mature leaf tissue from *Plantago* plants was frozen at − 80 °C and ground by stainless steel ball bearing for 30 s at 30 Hz using a MM400 Mixer Mill (Retsch, Germany) fitted with 2 ml tube adapter. DNA was extracted from ground leaf tissue following Healey et al.^44^. Nuclear ribosomal internal transcribed spacer (ITS) regions were amplified by PCR using primers^45^ and conditions listed in Supplementary Table 2. Amplified ITS regions were sequenced by AGRF (Adelaide, Australia). Where possible, sequence identities were confirmed by MUSCLE nucleotide alignment with published sequences from Rønsted et al.^46^ and Tay et al.^47^. The ITS sequence of *Veronica salicornioides*, FJ024624.1^47^, was downloaded from Genbank and used as an outgroup as per Rønsted et al.^46^.

ITS sequences were trimmed using BMGE^48^ and the maximum-likelihood tree constructed in Geneious v8.1.3 (Biomatters Ltd, NZ) with the RAxML tree builder^49^ using the GTR GAMMA nucleotide model with 500 rapid bootstrapping replicates.

### Seed morphometric measurements

Seed length and width measurements were determined by image analysis. Images of seeds were taken at 1 × magnification on an AxioImager M2 (Zeiss, Germany) fitted with an AxioCam 105 colour camera (Zeiss, Germany). Length and width of 20 seeds per species were measured using ZEN 2012 software (Zeiss, Germany). To determine 1000 seed weight, seeds were manually counted and weighed.

### Mucilage microscopy

Mucilage architecture was observed by whole mount immunolocalisation of heteroxylan using the LM11 antibody^50^ and ruthenium red staining following Phan et al.^33^. Ruthenium red used in this work (C075) was purchased from ProSciTech (Australia) and prepared as per Cowley et al.^51^.

### Whole seed thin section microscopy and immunolabelling

Whole seeds were transversely halved with a razor blade and fixed and embedded in LR White resin as per Burton et al.^52^ modified to use the 80% ethanol fixative described in Phan et al.^9^. Fixed and embedded tissue was sectioned at 1 µm on an Ultramicrotome (Leica, EM UC6) using a diamond knife (DiATOME, Nidau, Switzerland). After staining thin sections with toluidine blue, internal seed structures were imaged with an AxioImager M2 (Zeiss, Germany) fitted with an AxioCam 105 colour camera (Zeiss, Germany). Endosperm mannan polysaccharides were immunolocalised using a tenfold dilution of LM21 anti-(1,4)-β-mannan primary antibody (Kerafast, US). The secondary antibody, Alexa Fluor 555 goat anti-rat IgG, was applied at a 100-fold dilution. All fluorescent images were taken with a Zeiss M2 AxioImager with an AxioCam 506 mono black and white camera. Images were processed using ZEN 2012 software (Zeiss, Germany).

### Seed mucilage fractionation and yield analysis

Mucilage was extracted and fractionated and yield traits were determined following Cowley et al.^51^ with no deviation from the described procedure. Briefly, 1 mL of water was added to 30 mg of seed and extracted at 25 °C for 1.5 h with agitation. After brief centrifugation, the supernatant was transferred to a new tube (cold water extractable (CWE) mucilage fraction) and the volume of the pellet-containing tube was returned to 1 mL. The extraction was repeated again, but at 65 °C to obtain the hot water extractable (HWE) mucilage fraction. Finally, the pellet (seeds with the most extraction resistant mucilage fraction) were agitated on a tissue disruptor-type mill at 30 Hz for 10 min to obtain the intense agitation extractable (IAE) fraction. Collected fractions were freeze-dried to a constant weight and compared to starting mass of seeds to determine the yield:

$$\text{Yield}\,\left({\%}\right)=\left(\frac{\text{mass of freeze dried mucilage}}{\text{mass of seeds pre-extraction}}\right)\times 100$$

### Water absorption capacity assay

The water absorption capacity was determined following Cowley et al.^51^ with modifications. As the seeds were of variable size, 30 mg were weighed into 2 mL microcentrifuge tubes and 1 g of deionized water was added to each tube. After briefly vortexing to break surface tension and submerge seeds, the seed mucilage was allowed to expand, undisturbed, for 45 min at room temperature (25 °C). After 45 min, a 1000 μL laboratory pipette was used to remove unabsorbed water, avoiding disturbing the seeds and their mucilage. Removed water was weighed and the water absorption capacity was determined using the following equation:

$$\text{Water absorption capacity }\left(\text{g/g}\right)=\frac{\text{Initial weight of water added}-\text{weight of unabsorbed water}}{\text{Initial weight of seeds added}}$$

### Monosaccharide analysis

Monosaccharide profiles of fractionated mucilage (redispersed at 1 mg/mL in Milli-Q water) and WSF were determined using reverse phase high performance liquid chromatography (RP-HPLC) of 1-phenyl-3-methyl-5-pyrazoline (PMP) derivatives following Cowley et al.^51^. Area under the peaks was compared to standard curves of mannose, ribose, rhamnose, glucuronic acid, galacturonic acid, glucose, galactose, xylose, arabinose and fucose^53^.

### Whole seed soluble sugar extraction and profiling

Soluble sugars were extracted from 10 mg of WSF following Vespreet et al.^54^ with modifications based on Matros et al.^55^. Due to substantial mucilage gelling in water when attempting a sequential 80% ethanol and water extraction, soluble sugars were twice extracted from flour in 80% ethanol at 85 °C for 30 min on a mixer (700 rpm) at a final dilution of 1:40 (w/v, mg/µl). Supernatants were combined, diluted with water to 1:25,000 (w/v, mg/µl) and 25 µl per sample were analysed by high pH anion exchange chromatography with pulsed amperometric detection (HPAEC–PAD) on a Dionex ICS-5000 system using a DionexCarboPAC^TM^PA-20 column (3 × 150 mm) with a guard column (3 × 50 mm) kept at 30 °C and operated at a flow rate of 0.5 mL min^−1^. The eluents used were (A) 0.1 M sodium hydroxide and (B) 0.1 M sodium hydroxide with 1 M sodium acetate. The gradient used was: 0% (B) from 0 to 2 min, 20% (B) from 2 to 35 min, 100% (B) from 35 to 36.5 min, 0% (B) from 37.5–38.5 min. Detector temperature was maintained at 20 °C, data collection was at 2 Hz and the Gold Standard PAD waveform (std. quad. potential) was used.

Data acquisition, processing, and peak integration were performed using the Chromeleon™ version 7.1.3.2425 software (Thermo Scientific). Compounds were annotated and quantified based on peaks of available analytical standards. Glucose, fructose, sucrose, raffinose, 1-kestose, maltose, nystose and stachyose analytical standards were purchased from Sigma-Aldrich, while 1,1,1-kestopentaose was obtained from Bio-Strategy. A standard of planteose was purified and prepared from *Salvia hispanica* seed mucilage as per Xing et al.^56^. Schematic structures were prepared using the DrawGlycan-SNFG online tool ( http://www.virtualglycome.org/DrawGlycan/)^57^.

### Protein analysis

Protein content of 150 mg of WSF was determined by the Dumas method using a Rapid N Exceed bench top nitrogen analyser (Elementar, USA). Conversion factor was 6.25. Protein content was determined in triplicate.

### Lipid analysis

Total lipid in 500 mg of WSF was determined by modified Folch method^58^ and fatty acid profiles were determined by gas chromatography of transesterified lipids following Liu et al.^59^. Analyses were performed in duplicate at SAHMRI (Adelaide, Australia). Multivariate principle component analysis for separation was performed in PAST software (version 3.25)^60^.

### Energy calculation

Energy content of whole seeds was estimated from the energy density of protein, carbohydrate, dietary fibre and fat components in whole *Plantago* seed^61^. Average protein, carbohydrate, dietary fibre, and fat contents (% w/w) were obtained from whole seed protein, soluble sugar, monosaccharide, and lipid profiling analyses, respectively.

### Figures

Unless otherwise indicated, plots were generated using Prism 8.4 (GraphPad, USA).

### Consent for publication

All authors give consent for the data to be published.

## Results

### Genetic relationships

**Figure 1:**
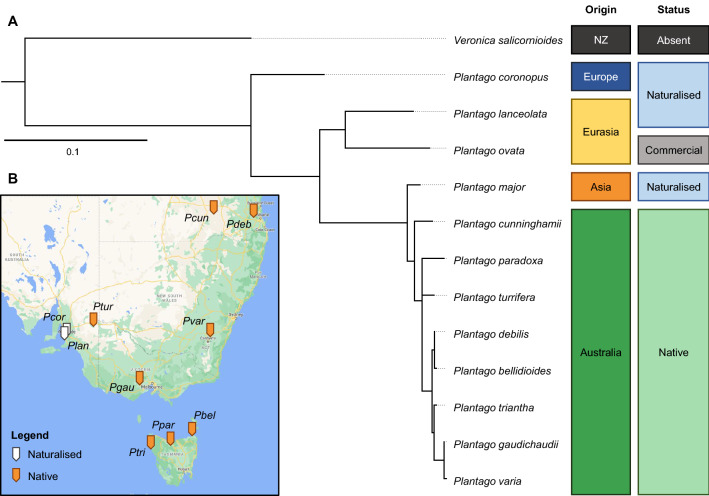
(**A**) Maximum-likelihood tree of nuclear ribosomal DNA internally transcribed spacer (ITS) regions of *Plantago* species studied here. The ITS sequence of *Veronica salicornioides*, a New Zealand (NZ)-endemic relative in Plantaginaceae, is included as the outgroup. Noted alongside each species are their geographic origin (where native to) and the status of occurrences in Australia (naturalised, native or grown commercially). Scale refers to substitutions per site. (**B**) Locations of *Plantago* sources in South Eastern Australia based on coordinates supplied with seedbank-withdrawn accessions or collection sites. Map sourced from Google Earth earth.google.com/web. *P. ovata* and *P. major* samples were purchased commercially, so origin information can only be assumed. Abbreviations: *Pbel* = *P. bellidioides*; *Pcor* = *P. coronopus*; *Pcun* = *P. cunninghamii*; *Pdeb* = *P. debilis*; *Pgau* = *P. gaudichaudii*; *Plan* = *P. lanceolata*; *Ppar* = *P. paradoxa*; *Ptri* = *P. triantha*; *Ptur* = *P. turrifera*; *Pvar* = *P. varia.*

A maximum-likelihood tree of nuclear ribosomal internally transcribed spacer (ITS) region sequences^46,47^ of the *Plantago* species studied here was used to produce a phylogenetic tree (Fig. 1). The species can be separated into clades by their origin, *P. coronopus* from Europe, *P. lanceolata* and *P. ovata* from Central Europe and Eurasia, and the remaining species from Australasia. The Australasian *Plantago* species, particularly Australian natives, are more genetically-similar than the other species studied here, but *P. major*, a naturalised species, is grouped into an Asian clade.

Using the tree in Fig. 1, the 12 species were ranked based on their relative genetic distance from the outgroup and each other and this order is used in all subsequent figures to allow observation of trends between more distantly and more closely-related species.

### Seed mucilage characteristics

**Figure 2:**
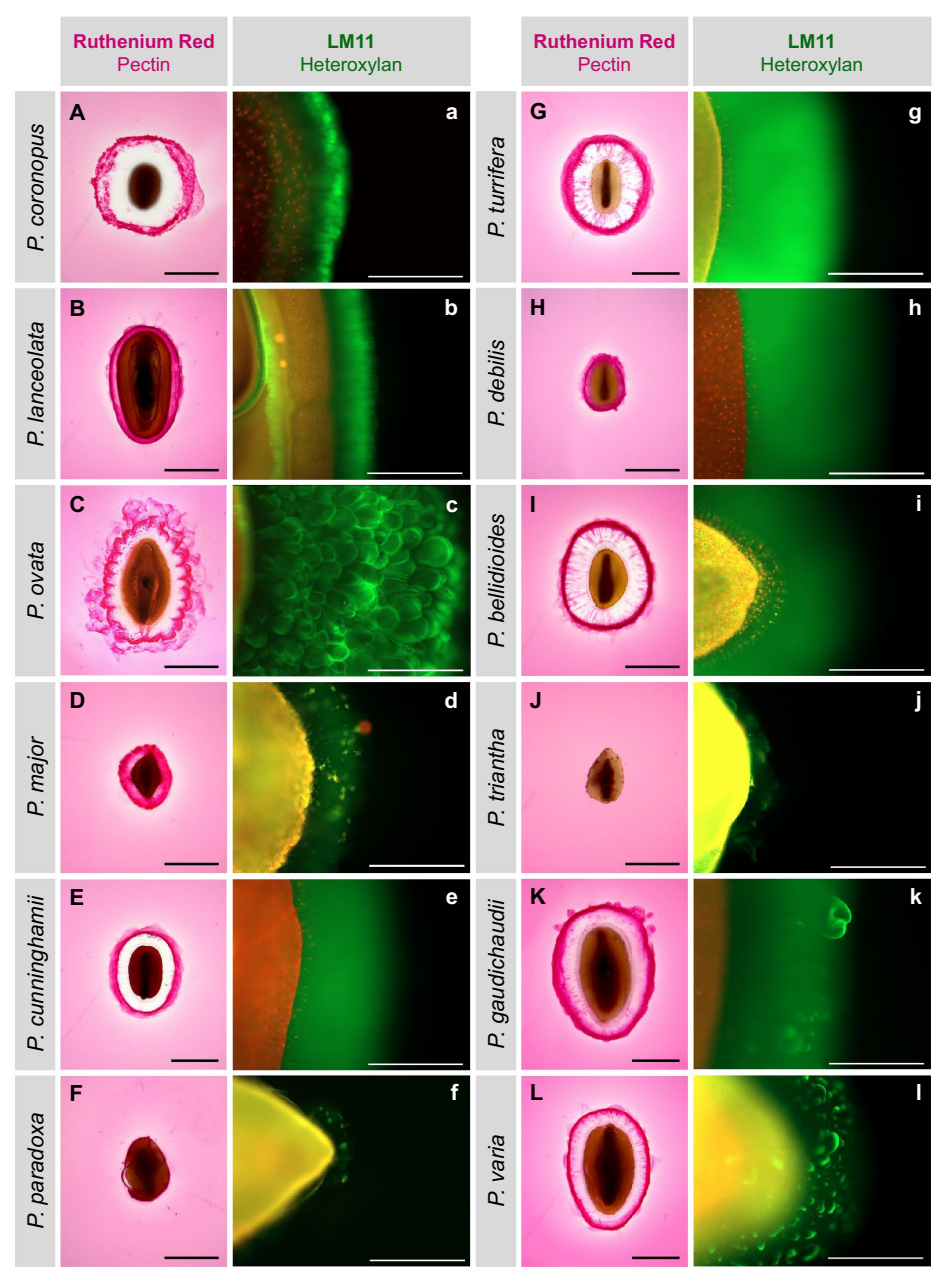
Expanded mucilage architecture of 12 *Plantago* species. Ruthenium red (dark pink) stains acidic polysaccharides at the mucilage periphery (**A–L**). Scale = 1 mm. Anti-xylan antibody LM11 (green) probes for xylan backbone epitopes in the inner mucilage layers (**a–l**) while the seed is counterstained with propidium iodide (red/yellow). Scale = 500 µm.

**Figure 3:**
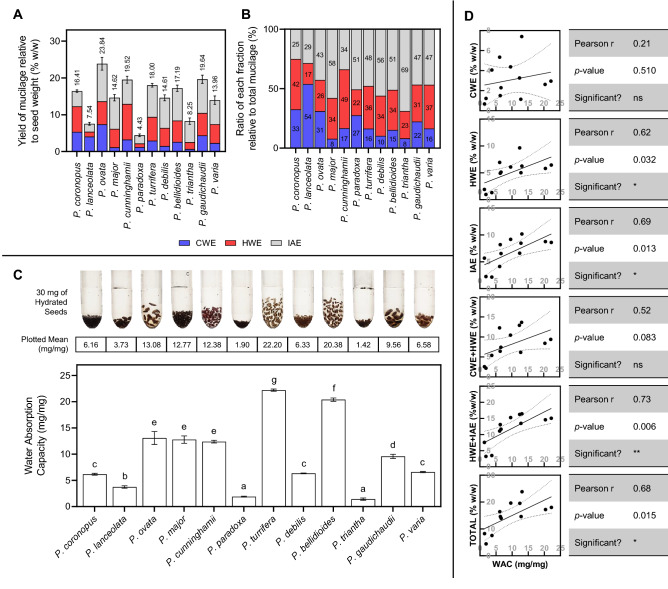
Seed mucilage yield, fractionation and water absorption traits of 12 *Plantago* species. (**A**) Yield of mucilage as a fraction of the whole seed. (**B**) Quantity of each isolated mucilage fraction as share of total mucilage. Values within bars are plotted values rounded to the nearest integer. CWE = cold water extractable (blue); HWE = hot water extractable (red); IAE = intense agitation extractable (grey). (**C**) Water absorption capacity of *Plantago* seeds. Above the plot are plotted mean values and a representative image of 30 mg of hydrated and swollen *Plantago* seeds. Samples sharing a letter are not significantly different from each other (*p* > 0.05). (**D**) Study of correlations between water absorption capacity (WAC) and mucilage yield/fractionation traits (seed-relative traits from **A**). Linear models plotted with 95% confidence bands. ns = not significant. Values presented are means of five replicates in (**A**) and (**B**) and three replicates in (**C**) Error bars correspond to one standard deviation.

There is significant variation in expanded seed mucilage architecture of the *Plantago* species studied here (Fig. 2). We corroborate the same characteristics and differences in ruthenium red phenotype of *P. coronopus, P. lanceolata, P. ovata, P. major, P. cunninghamii*, *P. debilis* and *P. varia* described in detail by Phan et al.^33^. Here we studied five additional Australian native species and found, owing to their relatedness (Fig. 1), *P. turrifera* and *P. bellidioides* to be strikingly similar to *P. cunninghamii* and *P. debilis,* and *P. gaudichaudii* to be most similar to *P. varia*. These similarities were also found when immunolabelling the mucilage (Fig. 2a–l). Two further native species, *P. paradoxa* and *P. triantha,* despite their apparent genetic similarity to other native species (Fig. 1), produced a negligible mucilage envelope that was poorly distinguishable with ruthenium red staining (Fig. 2F and 3J) and only slightly more apparent with xylan-directed immunolabelling (Fig. 2f and 3j).

In this study we used our previously published mucilage fractionation method^51^ to investigate mucilage yield and fractionation traits (Fig. 3). There was significant interspecific variation in total yield (Fig. 3A) and fractionation profile (Fig. 3B) of seed mucilage. Total yield of mucilage was highest in *P. ovata* and comparatively lower in *P. lanceolata, P. paradoxa,* and *P. triantha.* Australian native species, *P. cunninghamii, P. turrifera, P. bellidioides, P. debilis, P. gaudichaudii* and *P. varia* had quite similar total yield (Fig. 3A) and fractionation profiles (Fig. 3B) and less mucilage could be extracted using cold water (CWE). To complement mucilage extractability traits, the water absorption capacity by whole *Plantago* seeds was determined (Fig. 3C). Water absorption capacity (WAC) was found to vary substantially with *P. turrifera* and *P. bellidioides* having a significantly higher WAC than other species, while absorption by *P. lanceolata, P. paradoxa* and *P. triantha* was very low. A correlation study was performed to determine if there was a link between mucilage fractionation and yield traits and the observed differences in WAC (Fig. 3D). Logically, significant (*p* < 0.05) correlations between WAC and total mucilage content and WAC and the more gel-like extraction resistant mucilage fractions were found. The strongest correlation (r = 0.73, *p* = 0.006) with WAC was the proportion of hot water- and intense agitation extractable (HWE + IAE) mucilage.

**Figure 4:**
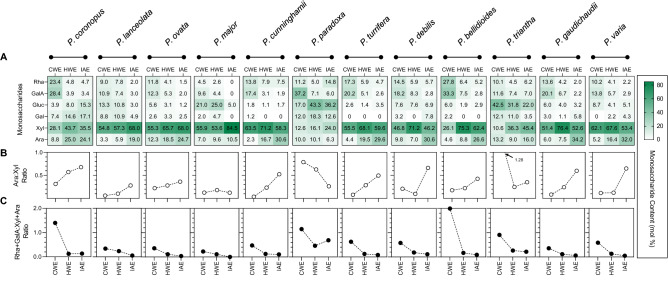
Chemical composition of fractionated mucilage from 12 *Plantago* species. (**A**) Monosaccharide composition of each mucilage fraction. Heatmap of relative (molar) abundance of each monosaccharide in a fraction. (**B**) Arabinose to xylose (Ara:Xyl) ratio in each fraction. (**C**) Pectin (rhamnose and galacturonic acid) to heteroxylan (xylose and arabinose) (Rha + GalA:Xyl + Ara) ratio in each fraction. CWE = cold water extractable; HWE = hot water extractable; IAE = intense agitation extractable. Values presented are the means of five fractionation preps.

The chemical composition of fractionated mucilage was determined by monosaccharide profiling which revealed both interspecific differences and trends (Fig. 4). Monosaccharide profiles were similar between all fractions of all species, with xylose and arabinose (constituents of heteroxylan) comprising the majority of monosaccharides quantified along with rhamnose and galacturonic acid (constituents of pectin) and minor quantities of glucose and galactose (Fig. 4A). Other monosaccharides for which standards were included, mannose, ribose, glucuronic acid, and fucose, were minute or not detected (data not shown). The relative abundance of each monosaccharide detected in the mucilage fractions, however, differed significantly. The arabinose to xylose ratio differed interspecifically and also between fractions. In almost all species, the arabinose to xylose ratio rises with increasingly harsh extraction steps (Fig. 4B). In *P. paradoxa*, the inverse was observed and in the CWE fraction of *P. triantha* the value was outside the expected range of less than 1 (1.28). These species produced little mucilage and displayed poor monosaccharide recovery which explains some of the discrepancies. In particular, the CWE mucilage fraction from *P. triantha* was very small and the detected arabinose and xylose are likely not heteroxylan-derived. Values for the more abundant HWE and IAE fractions are within the expected range (< 1). The ratio of rhamnose to galacturonic acid was stable between fractions and differed minimally between species (Fig. 4A). Rhamnose and galacturonic acid, however, were found to be highly-enriched in the CWE fraction of all species (Fig. 4C) but much lower or even absent in subsequent fractions.

### *Plantago* endosperm and embryo morphology and composition

**Figure 5:**
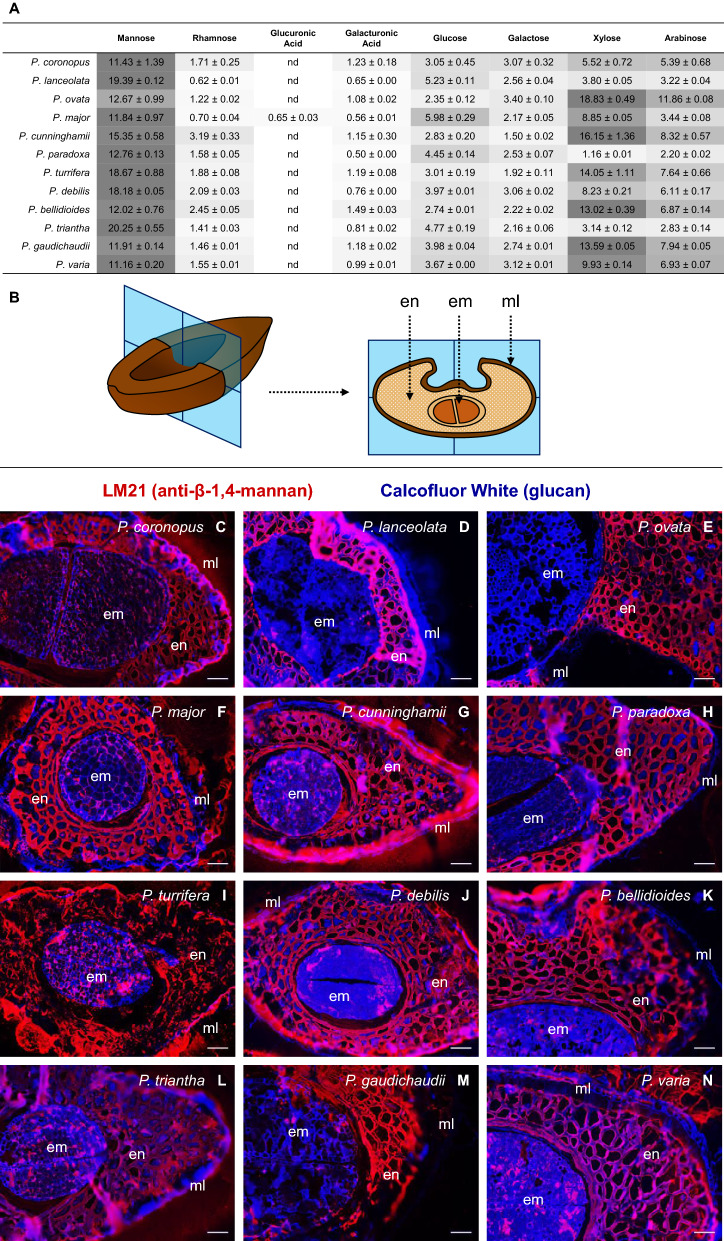
Profiling of the major polysaccharide sugars in *Plantago* seed. (**A**) Monosaccharide profiles of whole *Plantago* seeds (means of three replicates, % w/w, with standard deviation). A grey scale (dark grey, highest value; white, lowest value) has been used to highlight the most abundant monosaccharides in a species. (**B**) Schematic diagram of the orientation of thin sections taken from *Plantago* seeds. (**C–N**) Immunodetection of mannan in *Plantago* endosperm cell walls by LM21 monoclonal antibody. Scale = 50 μm. Abbreviations: en = endosperm; em = embryo; ml = mucilage layer.

Monosaccharide profiling revealed that mannose is a major non-cellulosic monosaccharide in whole seeds of all species, being the most abundant in 8 of 12 species studied here (Fig. 5A). Using a monoclonal antibody recognising β-(1,4)-mannan^62^ it was observed that *Plantago* species have a substantial endosperm with thick (grand median = 8.206 μm, Supplementary Fig. 1) mannan-rich walls (Fig. 5C–L). Minimal amounts of mannan were detected in the embryo but it was completely absent in the mucilage layer of all species except *P. cunninghamii* (Fig. 5G). However, mannose is completely absent in *P. cunninghamii* mucilage, even that tightly adhered to the seed^51^ suggesting that the mannan labelling seen in the mucilage layer of *P. cunninghamii* is likely an artefact of non-specific binding in poorly-fixed mucilage. The endosperm cells also all contained tightly-packed spherical bodies (Supplementary Fig. 2) which are likely to be aleurone grains and/or oil bodies, similar to those seen in seed tissues of other species like *Cannabis sativa*^63^.

### Soluble sugar profiling

**Figure 6:**
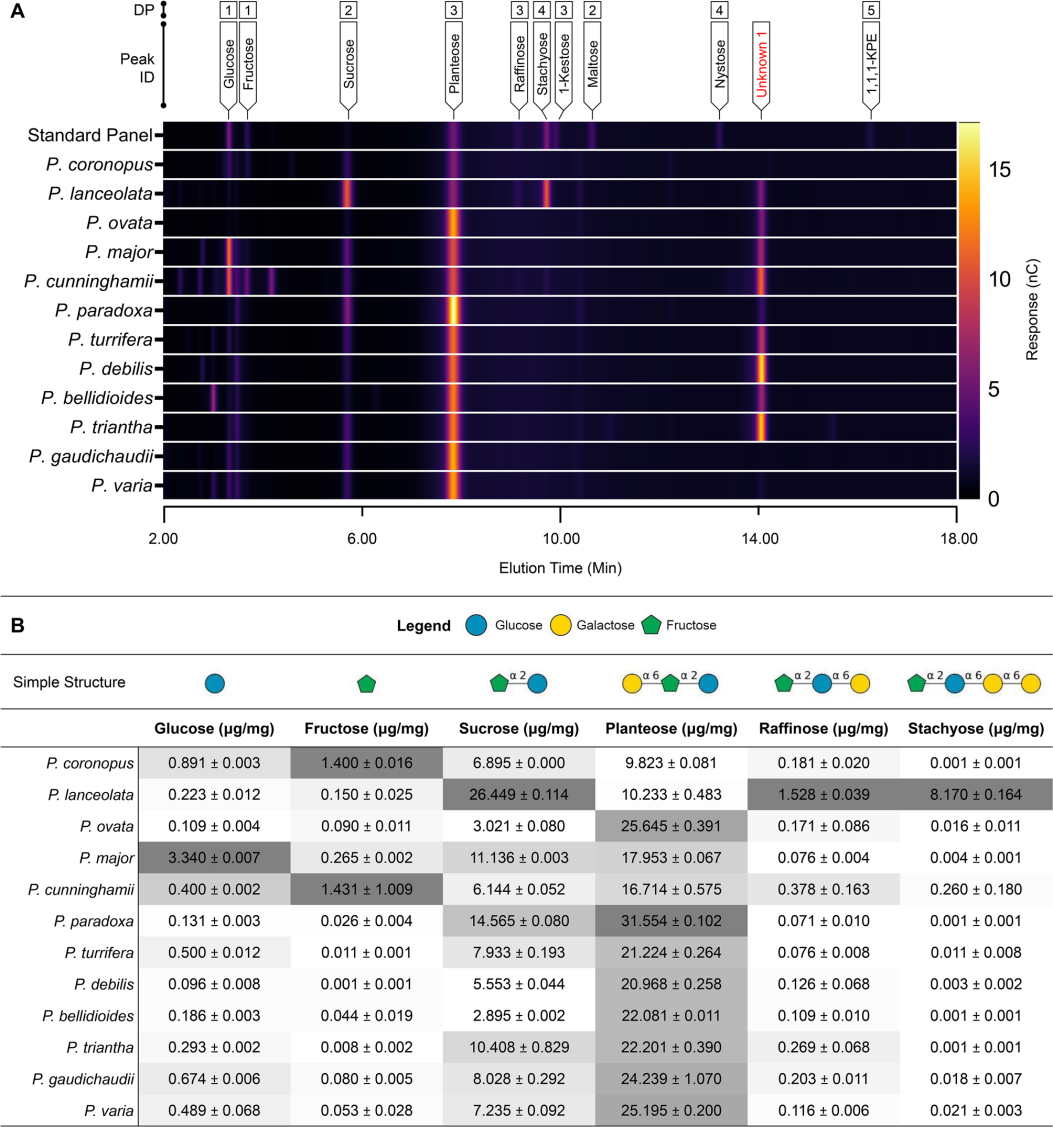
Soluble sugar profiling of *Plantago* seeds. (**A**) Chromatographic data presented in heatmap form to highlight differences in chromatographic profile between species. Chromatographic data has been cropped to an elution period that includes saccharides with a low degree-of-polymerisation (DP) (2–18 min). For simplicity, chromatographic data of standard runs (standard panel: a combined low DP saccharide suite, planteose and stachyose) were combined. (**B**) Quantification of key low DP saccharides and their schematic structure. A grey scale (dark grey, highest value; white, lowest value) has been used to display differences in abundance of one type between species. Values are means and standard deviation of three replicates. Abbreviations: 1,1,1-KPE = 1,1,1-Kestopentaose.

Soluble sugar profiling was used to determine the content of rapidly-mobile non-starch reserve carbohydrates in the internal *Plantago* seed tissues (Fig. 6). Heat mapped chromatographic data (Fig. 6A) show that the soluble sugar profiles are similar between all species with common major components, with most of the interspecific variation coming from differing abundances of the major sugars, sucrose (*β*-d-fructofuranosyl-(2 → 1)-*α*-d-glucopyranoside) and planteose (*α*-d-galactopyranosyl-(1 → 6)-*β*-d-fructofuranosyl-(2 → 1)-*α*-d-glucopyranoside) which account for up to 86% of the soluble sugar content (Supplementary Fig. 3). Some variation between profiles was evident between 2 and 5 min elution times but these components are (based on relative peak area; Supplementary Fig. 3) lowly abundant minor sugars, possibly unidentified mono- or di-saccharides. Quantification of extracted soluble sugars shows that the trisaccharide planteose is the major soluble sugar in seeds of the *Plantago* species studied here (up to 3.2% w/w), except in *P. lanceolata* where sucrose dominates, although followed by planteose (Fig. 6B). *P. lanceolata* also had the most unique soluble sugar profile in other ways: the trisaccharide raffinose (*β*-d-fructofuranosyl-(2 → 1)-*α*-d-glucopyranoside-(1 → 6)-*α*-d-galactopyranoside) and its higher homologue, the tetrasaccharide stachyose (*β*-d-fructofuranosyl-(2 → 1)-*α*-d-glucopyranoside-(1 → 6)-*α*-d-galactopyranosyl-(1 → 6)-*α*-d-galactopyranoside), which, present in minute quantities in other species, were additional major soluble sugars in *P. lanceolata*.

Currently unidentified, we also detected an additional sugar, Unknown 1 (Fig. 6A), eluting at approximately 14 min in extracts of all species. Based on the relative chromatographic peak area, Unknown 1 appears to be a major soluble sugar in seven of the species tested, comparable to planteose amounts in *P. debilis* or *P. triantha* (Supplementary Fig. 3). Levels of other oligosaccharides included in the standard panel, 1-kestose, maltose, nystose, and 1,1,1-kestopentaose were minute or not detected in the species studied here.

### Morphometric and nutritional characteristics of Plantago seeds

*Plantago* seeds differ significantly in length, width and thousand grain weight (Table 1) and can be categorised as small- or large-seeded (Supplementary Fig. 4). Seed size is not origin-specific as both native and non-native species are included in the small- and large-seeded categories (Supplementary Fig. 4).

**Table 1:** Summary of morphometric and nutritional characteristics of seeds of 12 *Plantago* species. Values are means and standard deviation.

		*P. coronopus*	*P. lanceolata*	*P. ovata*	*P. major*	*P. cunninghamii*	*P. paradoxa*
**Seed Morphometric Characteristics**							
Length (mm)		1.138 ± 0.132	2.679 ± 0.164	2.843 ± 0.224	1.144 ± 0.060	1.394 ± 0.111	1.556 ± 0.095
Width (mm)		0.740 ± 0.075	1.227 ± 0.143	1.448 ± 0.108	0.653 ± 0.081	0.822 ± 0.044	1.101 ± 0.087
1000 Grain Weight (mg)		205 ± 8	1508 ± 33	1618 ± 43	172 ± 5	364 ± 2	411 ± 17
**Seed Nutrient Composition**							
Energy (kJ/100 g)		1176.15	844.89	898.3	1064.17	1260.15	1239.47
Protein (%)		19.91 ± 2.34	11.20 ± 0.08	11.42 ± 1.63	16.36 ± 0.35	19.53 ± 0.20	31.35 ± 2.16
Carbohydrate (%)		1.92 ± 0.02	4.68 ± 0.15	2.91 ± 0.11	3.28 ± 0.01	2.63 ± 0.58	4.63 ± 0.04
Dietary Fibre (%)		31.4 ± 2.99	35.47 ± 0.26	51.41 ± 0.63	34.19 ± 1.13	48.49 ± 2.52	25.19 ± 0.31
Fat (%)		14.45 ± 0.47	7.76 ± 0.54	7.08 ± 0.58	12.70 ± 0.44	13.14 ± 1.07	10.41 ± 1.28
**Fatty Acid Composition of Seed Oil**							
**Saturated**	Total (%)	16.85 ± 0.16	22.39 ± 0.37	17.20 ± 0.14	14.37 ± 1.33	15.01 ± 0.03	15.76 ± 0.02
Myristic Acid (14:0) (%)		0.04 ± 0.01	0.06 ± 0.01	0.06 ± 0.03	0.05 ± 0.02	0.09 ± 0.02	0.11 ± 0.00
Pentadecylic Acid (15:0) (%)		0.08 ± 0.01	0.17 ± 0.01	0.12 ± 0.01	0.05 ± 0.02	0.05 ± 0.00	0.07 ± 0.02
Palmitic Acid (16:0) (%)		12.05 ± 0.15	17.52 ± 0.17	12.43 ± 0.13	10.23 ± 0.90	11.8 ± 0.11	11.84 ± 0.06
Margaric Acid (17:0) (%)		0.10 ± 0.01	0.13 ± 0.02	0.11 ± 0.01	0.15 ± 0.00	0.06 ± 0.01	0.09 ± 0.01
Stearic Acid (18:0) (%)		3.83 ± 0.03	3.70 ± 0.10	3.82 ± 0.06	2.82 ± 0.27	2.62 ± 0.17	3.11 ± 0.03
Arachidic Acid (20:0) (%)		0.50 ± 0.01	0.45 ± 0.05	0.42 ± 0.00	0.57 ± 0.06	0.26 ± 0.00	0.38 ± 0.01
Behenic Acid (22:0) (%)		0.17 ± 0.02	0.21 ± 0.01	0.19 ± 0.03	0.39 ± 0.04	0.12 ± 0.01	0.16 ± 0.01
Lignoceric Acid (24:0) (%)		0.09 ± 0.01	0.15 ± 0.03	0.05 ± 0.06	0.11 ± 0.03	0.03 ± 0.04	0.00 ± 0.00
**Unsaturated**	Total (%)	82.99 ± 0.02	77.53 ± 0.48	82.76 ± 0.08	85.53 ± 1.41	84.92 ± 0.01	84.24 ± 0.02
**ω-3**	Total (%)	27.98 ± 0.02	6.74 ± 0.22	3.18 ± 0.12	12.31 ± 1.70	43.42 ± 0.03	32.09 ± 0.12
Alpha-Linolenic Acid (ALA) (18:3) (%)		27.98 ± 0.02	6.74 ± 0.22	3.18 ± 0.12	12.31 ± 1.70	43.42 ± 0.03	32.09 ± 0.12
**ω-6**	Total (%)	38.29 ± 0.09	43.99 ± 0.36	39.72 ± 0.29	52.30 ± 1.68	16.60 ± 0.20	23.38 ± 0.13
Linoleic Acid (18:2) (%)		38.22 ± 0.09	43.95 ± 0.42	39.65 ± 0.31	52.21 ± 1.69	16.57 ± 0.21	23.34 ± 0.19
Eicosadienoic Acid (20:2) (%)		0.06 ± 0.01	0.04 ± 0.06	0.07 ± 0.02	0.09 ± 0.01	0.03 ± 0.01	0.04 ± 0.06
**ω-7**	Total (%)	0.76 ± 0.01	1.73 ± 0.06	1.37 ± 0.03	1.10 ± 0.05	1.44 ± 0.04	1.16 ± 0.01
Palmitoleic Acid (16:1) (%)		0.08 ± 0.00	0.23 ± 0.02	0.16 ± 0.02	0.10 ± 0.01	0.21 ± 0.00	0.18 ± 0.02
Vaccenic Acid (18:1) (%)		0.67 ± 0.01	1.49 ± 0.04	1.20 ± 0.05	0.99 ± 0.06	1.23 ± 0.04	0.99 ± 0.00
**ω-9**	Total (%)	15.96 ± 0.10	25.08 ± 0.15	38.49 ± 0.47	19.82 ± 1.91	23.46 ± 0.21	27.60 ± 0.29
Oleic Acid (18:1) (%)		15.83 ± 0.11	24.90 ± 0.13	38.12 ± 0.49	19.57 ± 1.88	23.35 ± 0.20	27.49 ± 0.34
Gondoic Acid (20:1) (%)		0.13 ± 0.01	0.18 ± 0.01	0.36 ± 0.02	0.26 ± 0.02	0.12 ± 0.02	0.12 ± 0.05
**ω-3:ω-6**		0.76 ± 0.04	0.16 ± 0.01	0.11 ± 0.04	0.28 ± 0.09	2.64 ± 0.06	1.37 ± 0.00
**Yield of ALA**	(% w/w of whole seed)	4.04 ± 0.13	0.52 ± 0.02	0.23 ± 0.01	1.56 ± 0.16	5.71 ± 0.46	3.34 ± 0.40

		*P. turrifera*	*P. debilis*	*P. bellidioides*	*P. triantha*	*P. gaudichaudii*	*P. varia*
**Seed Morphometric Characteristics**							
Length (mm)		1.562 ± 0.044	1.308 ± 0.071	1.563 ± 0.115	1.236 ± 0.132	2.820 ± 0.292	2.495 ± 0.250
Width (mm)		0.840 ± 0.086	0.717 ± 0.036	0.879 ± 0.079	0.818 ± 0.07	1.490 ± 0.134	1.387 ± 0.142
1000 Grain Weight (mg)		355 ± 6	288 ± 9	383 ± 8	274 ± 4	1936 ± 102	1761 ± 24
**Seed Nutrient Composition**							
Energy (kJ/100 g)		1174.72	1109.7	1162.47	1142.19	1209.43	1190.19
Protein (%)		18.03 ± 1.06	13.80 ± 0.07	20.43 ± 0.22	30.92 ± 0.35	24.05 ± 1.35	26.01 ± 0.80
Carbohydrate (%)		2.98 ± 0.10	2.67 ± 0.08	2.53 ± 0.0	3.32 ± 0.26	3.32 ± 0.28	3.31 ± 0.04
Dietary Fibre (%)		48.36 ± 2.33	42.39 ± 0.35	40.80 ± 0.10	35.38 ± 0.84	42.8 ± 0.05	37.34 ± 0.32
Fat (%)		11.51 ± 0.81	12.90 ± 1.44	12.07 ± 0.04	7.48 ± 1.78	8.80 ± 0.84	9.11 ± 0.47
**Fatty Acid Composition of Seed Oil**							
**Saturated**	Total (%)	14.37 ± 0.67	18.73 ± 0.06	15.71 ± 0.11	22.14 ± 0.21	13.44 ± 0.45	16.64 ± 0.17
Myristic Acid (14:0) (%)		0.10 ± 0.02	0.07 ± 0.00	0.09 ± 0.00	0.09 ± 0.03	0.11 ± 0.03	0.14 ± 0.01
Pentadecylic Acid (15:0) (%)		0.05 ± 0.01	0.04 ± 0.00	0.06 ± 0.01	0.17 ± 0.00	0.05 ± 0.00	0.05 ± 0.00
Palmitic Acid (16:0) (%)		11.59 ± 0.45	14.18 ± 0.12	12.64 ± 0.08	17.59 ± 0.32	9.99 ± 0.32	12.84 ± 0.16
Margaric Acid (17:0) (%)		0.09 ± 0.02	0.08 ± 0.00	0.08 ± 0.01	0.13 ± 0.02	0.11 ± 0.01	0.12 ± 0.01
Stearic Acid (18:0) (%)		2.07 ± 0.18	3.63 ± 0.02	2.18 ± 0.08	3.46 ± 0.29	2.62 ± 0.13	2.85 ± 0.04
Arachidic Acid (20:0) (%)		0.24 ± 0.00	0.41 ± 0.02	0.30 ± 0.02	0.40 ± 0.00	0.35 ± 0.01	0.34 ± 0.01
Behenic Acid (22:0) (%)		0.12 ± 0.01	0.19 ± 0.01	0.22 ± 0.03	0.22 ± 0.06	0.13 ± 0.00	0.18 ± 0.04
Lignoceric Acid (24:0) (%)		0.10 ± 0.00	0.13 ± 0.03	0.15 ± 0.03	0.09 ± 0.12	0.09 ± 0.02	0.11 ± 0.02
**Unsaturated**	Total (%)	85.58 ± 0.59	81.19 ± 0.01	84.24 ± 0.04	77.80 ± 0.30	86.43 ± 0.33	83.22 ± 0.03
**ω-3**	Total (%)	54.46 ± 0.71	41.04 ± 0.06	41.35 ± 0.19	7.69 ± 1.08	46.23 ± 0.29	42.69 ± 0.95
Alpha-Linolenic Acid (ALA) (18:3) (%)		54.46 ± 0.71	41.35 ± 0.19	41.35 ± 0.19	7.69 ± 1.08	46.23 ± 0.29	42.69 ± 0.95
**ω-6**	Total (%)	11.30 ± 0.16	24.17 ± 0.10	18.67 ± 0.01	45.70 ± 1.81	15.93 ± 0.14	19.48 ± 0.61
Linoleic Acid (18:2) (%)		11.26 ± 0.15	18.63 ± 0.01	18.63 ± 0.01	45.65 ± 1.74	15.91 ± 0.11	19.44 ± 0.60
Eicosadienoic Acid (20:2) (%)		0.04 ± 0.01	0.04 ± 0.00	0.04 ± 0.00	0.05 ± 0.07	0.02 ± 0.03	0.04 ± 0.01
**ω-7**	Total (%)	1.66 ± 0.02	1.03 ± 0.00	1.31 ± 0.02	1.73 ± 0.03	1.15 ± 0.04	1.14 ± 0.03
Palmitoleic Acid (16:1) (%)		0.26 ± 0.02	0.15 ± 0.01	0.15 ± 0.01	0.26 ± 0.02	0.15 ± 0.01	0.14 ± 0.02
Vaccenic Acid (18:1) (%)		1.40 ± 0.01	1.17 ± 0.01	1.17 ± 0.01	1.47 ± 0.01	1.00 ± 0.02	1.00 ± 0.01
**ω-9**	Total (%)	18.15 ± 0.07	14.96 ± 0.15	22.90 ± 0.26	22.68 ± 3.17	23.13 ± 0.06	19.91 ± 0.34
Oleic Acid (18:1) (%)		18.07 ± 0.08	22.74 ± 0.25	22.74 ± 0.25	22.52 ± 3.13	23.00 ± 0.06	19.77 ± 0.33
Gondoic Acid (20:1) (%)		0.08 ± 0.01	0.16 ± 0.01	0.16 ± 0.01	0.16 ± 0.04	0.13 ± 0.01	0.14 ± 0.00
**ω-3:ω-6**		4.85 ± 0.09	1.70 ± 0.01	2.24 ± 0.04	0.17 ± 0.01	2.90 ± 0.01	2.22 ± 0.15
**Yield of ALA**	(% w/w of whole seed)	6.27 ± 0.52	5.29 ± 0.58	4.99 ± 0.00	0.58 ± 0.22	4.07 ± 0.42	3.88 ± 0.12

To determine if there is any nutritional value in *Plantago* seeds that is normally overlooked, the seed nutrient (protein, carbohydrate, dietary fibre, and fat) composition was determined (Table 1). There is a significant amount of interspecific variation in the content of each nutrient. Compared to commercial psyllium (*P. ovata*) which lies at the extremes in three of four nutrients (Supplementary Fig. 5), the other species studied have less dietary fibre but more fat and more protein, with native species tending to have the highest protein content. In carbohydrate content, *P. ovata* is close to the median. *P. ovata* and *P. lanceolata* have a lower energy content than all other species due to their low protein and fat content (~ 900 kJ vs. ~ 1100 kJ).

After determining that *Plantago* seeds contain a modest fat content, we investigated the fatty acid composition of the seed oil (Table 1). The ratio of saturated (SFA) to unsaturated fatty acids (UFA) is relatively similar between species (Median = 1:5.2) but six of eight native species were slightly higher in UFA content, up to 33% more than *P. ovata* in *P. gaudichaudii* (1:6.4). The saturated fatty acid profiles are very similar between species with palmitic (16:0) and stearic acid (18:0) accounting for at least 94% of SFA species. The most variation in fatty acid profiles comes from differences in the UFA profile. Multivariate analysis shows that 89% of variance between the species is due to differences in the ratio of omega-3 to omega-6 UFAs (Supplementary Fig. 6). All native species except *P. triantha* separate from commercial psyllium and the naturalised species because they contain a much higher quantity of omega-3 UFA in the seed oil; a significant difference between the non-native and native groups (*p* = 0.008, Supplementary Table 3). Commercial psyllium seed contains the least omega-3 UFA (3.2% w/w) of the species studied followed by the naturalised species (at most 28% w/w in *P. coronopus*). This is in clear contrast to the native species which contain an average of 39% omega-3 UFA (w/w), up to 54.5% in *P. turrifera*. When calculated as the omega-3 to omega-6 ratio, all native species have ratios greater than 1 (1.37–4.85) except *P. triantha* which has a very low ratio similar to non-native species which are all less than 1 (0.11–0.76). The yield of the major omega-3 UFA, alpha-linolenic acid (18:3) is particularly high in native species where, again, *P. turrifera* has the highest yield of 6.27% (w/w). There is also a small amount of variation (8%) from differences in the omega-6 to omega-9 UFA ratio. The omega-6 to omega-9 ratio was fairly similar between native species and much greater in naturalised species (up to 2.6 times greater than the native species average). Transaturated fatty acids were not present (data not shown).

## Discussion

In this study we present a comprehensive profiling of major nutritional and functional components of mucilage-producing seeds of 12 *Plantago* species that grow in Australia.

Mucilage comprises a major part of *Plantago* seeds and is the main factor currently influencing its use as a food additive and digestive health ingredient. Natural variation in *Plantago* mucilage properties is apparent even in the expanded mucilage architecture (Fig. 2). When extracting the seed mucilage in sequential fractions there are significant differences in WAC, yield and resistance to extraction (Fig. 3) and such variation may be explained by compositional differences (Fig. 4). The most easily-extracted *Plantago* seed fraction (CWE) contains abundant pectic monosaccharides, rhamnose and galacturonic acid, which are minimal in subsequent fractions (Fig. 4C). This is in line with our previous findings which suggest that the pectin in highly hydrophilic first mucilage fractions acts to ‘prime’ and initiate mucilage expansion^9^. Subsequent mucilage fractions which are obtained using more intense extraction conditions contain mostly xylose and arabinose, constituents of heteroxylan, in varying molar ratios. Yu et al.^64^ showed that the innermost layer(s) of mucilage in *P. ovata* was most gel-like as a result of high heteroxylan substitution complexity, corroborated in our previous publication^9^ and by others^65,66^. Here, we show the same trends in other *Plantago* species where the second and third (HWE and IAE) mucilage fractions (akin to the inner layers described by Yu et al.^64^) had the highest AX ratios, an indication of heteroxylan substitution complexity. Similarly, the HWE and IAE mucilage fractions of *P. cunninghamii*, *P. turrifera*, *P. bellidioides*, *P. debilis*, *P. gaudichaudii* and *P. varia* had particularly high AX ratios suggesting even more complex heteroxylan substitution (Fig. 3B), as indicated by minimal immunolabelling by LM11 because the unsubstituted/lowly-substituted backbone epitope is not present (Fig. 2e, g, h, i, k, and l). The inner mucilage fractions in these species also represented a large proportion of the total mucilage (Fig. 3B) and analysis of the WAC of mucilage on intact seeds (Fig. 3C) found WAC to be strongly correlated with both HWE (r = 0.62, *p* = 0.032) and IAE content (r = 0.69, *p* = 0.013) but the strongest correlation was a combination of the two (r = 0.73, *p* = 0.006) (Fig. 3D). Furthermore, the mucilage of these species is still apparent as retained, swollen envelopes even after extraction (compared to *P. lanceolata,* for example) (Supplementary Fig. 7) showing that the robustness of seed mucilage in Australian *Plantago* species is likely due, at least in part, to heteroxylan structure and resultant intermolecular bonds. It should be noted however that mucilage characteristics and composition appear to be at least partly independent of phylogenetic lineage and non-native/native status (*p* > 0.05, Supplementary Table 3). Instead, preliminary findings associating mucilage content and water absorption capacity with key environmental factors (Supplementary Fig. 8) suggest that these strong macromolecular properties may have developed to ensure germination success in climatically different Australian environments, as has already been demonstrated for *Plantago*^67^ and other species^68,69^. Importantly, the strong macromolecular properties in some Australian *Plantago* species like *P. turrifera* or *P. bellidioides* may also give them improved functionality over commercial psyllium in food and health applications where strong rheological/viscous properties are advantageous.

Along with the seed mucilage, we have also shown here that the internal tissues of *Plantago* seeds contain a wide array of saccharide families that represent dietary fibres that are typically underutilised during commercial psyllium production. After a review of the literature, we found that while *Plantago* species are known to have a substantial endosperm^70,71^, its exact composition has not been defined. The hard endosperm cell walls of *P. ovata* were suggested to be comprised solely of reserve cellulose^72^ but crystalline cellulose levels were found to be, at most, 4% (w/w)^73^. A clue is found during *P. ovata* seed development where mannan synthases are highly expressed along with the accumulation of mannose monosaccharides^74^. Here we are the first to confirm that the endosperm of all *Plantago* species studied contains thickened cell walls that are rich in mannan (Fig. 5). The substantial endosperm cell walls, like other species^75^, gives *Plantago* a ‘hard-seeded’ quality^76,77^ (for example, seeds of *P. ovata* require over four times the specific grinding energy compared to wheat^42,78^) but also results in a larger proportion of dietary fibre carbohydrates in the absence of starch^73^. In addition to the abundant endosperm polysaccharides, soluble sugars are also abundant in the *Plantago* seeds studied here (Fig. 6), in agreeance with previous reports^79^. Planteose (originally isolated from *Plantago* and named for the genus^80^) is the major soluble sugar in seeds of all *Plantago* species studied here except *P. lanceolata* where only sucrose is higher. While minute quantities of the oligosaccharides raffinose and stachyose were detected in all species, these sugars are only abundant in *P. lanceolata* seeds where stachyose levels were even similar to planteose. This agrees with previous findings that raffinose and stachyose were only detected in seeds of *Plantago* Sect. *Arnoglossum* of which *P. lanceolata* is the only member included here^81^. An additional sugar, Unknown 1 (Fig. 6A), was also found in all species. Based on relative chromatographic peak area, Unknown 1 appears to be a major soluble sugar in seven of the species tested, with levels even comparable to planteose in *P. debilis* or *P. triantha* (Supplementary Fig. 3). As the chromatographic method used here elutes soluble sugars roughly by their degree-of-polymerisation (DP), it is likely that Unknown 1 is a tetra- or pentasaccharide (DP4–5) as it elutes after nystose (DP4) but before 1,1,1-kestopentaose (DP5). While the exact identity of this compound will be elucidated by mass spectrometry, we hypothesise that Unknown 1 is likely to be the tetrasaccharide sesamose (*α*-d-galactopyranosyl-(1 → 6)-*α*-d-galactopyranosyl-(1 → 6)-*β*-d-fructofuranosyl-(2 → 1)-*α*-d-glucopyranoside), a higher DP homologue of planteose, which while not reported previously for *Plantago,* is found in tandem with planteose in other species^82^. The array of soluble sugars and mannan polysaccharides in internal tissues of *Plantago* seeds are likely to be the primary carbon source fuelling germination^83,84^, however they also have medicinal and nutritional significance as fermentable dietary fibres. Fermentable dietary fibres are consumed by beneficial bacteria in the human gut, producing short chain fatty acids like butyrate which is required to maintain colon health^85^ and there is also mounting evidence that modulating the microbiome through dietary fibre supplementation has significant effects on a wide range of disease indications (for review see Lynch & Pedersen^86^). While *P. ovata* husk is not a fermentable dietary fibre and thus provides limited fermentation- and microbiome-related benefits^87^, whole *P. ovata* seeds are reported to be fermentable, increasing faecal butyrate content in a clinical trial^88^ and improving numerous digestibility markers compared to the husk in a pre-clinical study^89^. These findings demonstrate that at least one non-husk component in *Plantago* seeds is fermentable by gut microbiota. While its exact prebiotic properties are currently unknown^56^, planteose, along with its homologues and other soluble sugars in *Plantago* seeds are likely fermentation candidates as many oligosaccharides are readily fermentable by beneficial bacteria in the gastrointestinal tract^90,91^. Endosperm mannan is also possibly fermentable compared to husk as diverse mannans and mannooligosaccharides released from mannan digestion have been shown to be well-fermented by a human faecal inoculum^92,93^.

Within the cells of the endosperm are large aleurone grains and oil bodies which house the protein and fat content of the *Plantago* seeds. Protein levels are generally similar to previous reports of *Plantago* species (10–20% w/w)^42,94–96^, but Australian *Plantago* species are generally higher compared to these reports and all are higher than commercial psyllium (*P. ovata*) (Table 1). *P. paradoxa* and *P. triantha* are very high in protein content, over 30% (w/w), which is higher than important grain legumes like chickpeas or lentil (24% and 26.1%, respectively)^97^. *Plantago* protein has previously been reported to contain essential amino acids and is well digested^95^ showing the potential of *Plantago* seeds as a protein source. Seeds of *Plantago* species are also rich in fats and all Australian species contain more fat than commercial psyllium, *P. ovata* (Table 1). Of particular nutritional importance is the proportion of omega-3 and omega-6 UFAs which mammals are unable to synthesise and are thus essential. There is a growing body of evidence that omega-3 fatty acids, particularly long-chain fatty acids (LCFAs) EPA and DHA commonly obtained from consuming fish, are protective against cardiovascular diseases^98^. However many researchers conclude that the world’s ecosystems could not sustainably supply the population with enough fish to provide the recommended EPA and DHA intake^99^ and thus sufficient intake of plant-derived UFAs to allow endogenous LCFA production from these precursors is recommended as an alternative. As plant-derived omega-3 (anti-inflammatory) and omega-6 (pro-inflammatory)^100,101^ UFAs are competitively desaturated and elongated into LCFAs by the same pathways, it is important that the ratio of omega-3 to omega-6 is at the very least 1:1^102^ to reduce the risk of inflammation. However an omega-3 to omega-6 intake ratio of 4:1 is suggested to be ideal for promoting heart, liver and gut health^103^. Here we show that Australian native *Plantago* seeds have omega-3 to omega-6 ratios greater than the minimum adequate ratio of 1:1 (Table 1) and after extensive review of the literature we report that *P. turrifera* has the highest ratio of omega-3 to omega-6 fatty acids reported for any seed (4.85:1). This is higher than benchmark species *Linum usitatissimum* (flax) or *Salvia hispanica* (chia), with 3.44:1 and 3.08:1, respectively^104^, although total fat content was 50% and 30% lower in *P. turrifera* than these species, respectively. Consumption of *Plantago* seeds, particularly those of Australian natives like *P. turrifera*, may therefore contribute to improving the undesirable omega-3 to omega-6 ratio that is common in modern, western diets, and thus diminish the associated health risks^100,102^.

## Conclusions and future directions

In this study we have provided a more complete understanding of the composition and morphology of seeds of twelve *Plantago* species that grow in Australia and outline implications these differences may have for food and nutritional applications. We have shown that among the species studied here there is variation in seed mucilage content and macromolecular properties with some native species having a higher capacity to absorb water than commercial psyllium, a key predictor of polysaccharide properties. We hypothesise that these differences are a result of fraction- and species-specific differences in heteroxylan and pectin content/structure and may represent wide differences in hydrocolloid functionality that can be exploited in industry. By profiling the nutrient content of the inner *Plantago* seed tissues (endosperm and embryo), we show that current commercial production of psyllium in particular underutilises the nutritional value of whole *Plantago* seeds. The use of a whole seed *Plantago* product, particularly from those species with favourable mucilage properties, and that are native and adapted to harsh Australian conditions, may provide a more nutritious but still functional alternative to commercial psyllium husk. In this way, the use of a *Plantago* WSF could confer multiple benefits: (1) hydrophilic mucilage polysaccharides could rheologically modify food systems and improve their quality; (2) minimal fermentation of the mucilage polysaccharides could benefit metabolic and gastrointestinal disorders that are improved when the viscosity and consistency of the gut digesta is increased; and (3) fermentable fibres and lipids along with other phytonutrients derived from the internal seed tissues could contribute to the improvement of a wide range of microbiome-, inflammatory- and oxidative stress-related disorders.

Future work will investigate how the specific differences in composition and properties outlined here impact the functionality of *Plantago* WSFs in food technology and gut health and aim to unravel the drivers of natural variation. The differences in seed mucilage content and properties appear to be mostly independent of phylogenetic lineage, however preliminary data suggests that differences among Australian native *Plantago* species might have developed as an ‘adaptive trait’ to cope with water-deficit stress in arid Australian environments suggesting that climate might be an important factor. Future work will aim to validate these preliminary findings by scrutinising more *Plantago* accessions that encompass more of the genus’ environmental range and provide insight into the intraspecific heterogeneity in the traits tested here. Probing this ‘adaptive trait’ hypothesis may aid in prospecting for ideally-adapted *Plantago* accessions with specific seed compositions for development as novel crops with tailored industrial end-uses.

## Supplementary Information

Supplementary Information.

## Data Availability

The datasets used and analysed during this work are available from the corresponding author upon reasonable request.
