# Supplementary material for: The composition of Australian Plantago seeds highlights their potential as nutritionally-rich functional food ingredients
Source: Sci Rep. 2021 Jun 16;11:12692. doi: 10.1038/s41598-021-92114-1 (PMC8209032; doi:10.1038/s41598-021-92114-1)
Supplement: Supplementary file 1 — Supplementary Information. [file 41598_2021_92114_MOESM1_ESM.docx]

**Supplementary File**

**The composition of Australian *Plantago* seeds highlights their potential as nutritionally-rich functional food ingredients**

**AUTHORS:** James M. Cowley^1,2^, Lisa A. O’Donovan^2^, and Rachel A. Burton^1,2*^

**AFFILIATIONS:** ^1^Australian Research Council Centre of Excellence in Plant Cell Walls, School of Agriculture, Food and Wine, University of Adelaide, Waite Campus, Urrbrae, SA, Australia; ^2^Australian Research Council Centre of Excellence in Plant Energy Biology, School of Agriculture, Food and Wine, University of Adelaide, Waite Campus, Urrbrae, SA, Australia;

* Author for correspondence: [rachel.burton@adelaide.edu.au](mailto:rachel.burton@adelaide.edu.au)

| **Species** | |  | **Origin** | **Seedbank** | **Voucher Number** | **ITS Genbank Accession No.** |
| --- | --- | --- | --- | --- | --- | --- |
| *P. coronopus* | C | | Austral Herbs, NSW, Australia | - | JMC001 | MW139232 |
| *P. lanceolata* | C | | Panorama, SA, Australia | - | JMC004 | MW139236 |
| *P. ovata* | P | | Austral Herbs, NSW, Australia | - | JMC006 | MW139238 |
| *P. major* | P | | Organics Australia, Australia | - | JMC005 | MW139237 |
| *P. cunninghamii* | R | | St. George, Queensland, Australia | - | JMC002 | MW139233 |
| *P. paradoxa* | S | | Central Plateau, Tasmania, Australia | TSCC | 0008866-a | MW139239 |
| *P. turrifera* | C | | Loxton, South Australia, Australia | - | JMC007 | MW139241 |
| *P. debilis* | R | | Charlwood, Queensland, Australia | - | JMC003 | MW139234 |
| *P. bellidioides* | S | | Petal Point, Tasmania, Australia | TSCC | 0008349-a | MW139231 |
| *P. triantha* | S | | Trial Harbour, Tasmania, Australia | TSCC | 0009460-a | MW139240 |
| *P. gaudichaudii* | S | | Mount Meredith, Victoria, Australia | VCS | MEL 2356648A | MW139235 |
| *P. varia* | R | | Aranda Bushland, Australian Capital Territory, Australia | - | JMC008 | MW139242 |

**Supplementary Table 1**. Sources for all *Plantago* species used in this study. Single letters denote material origin: P, purchased; C, private land collected; R, collected and donated by researcher; S, seedbank withdrawal. ALA = Atlas of Living Australia. TSCC = Tasmanian Seed Consveration Centre. VCS = Victorian Conservation Seedbank. ANBG = Australian National Botanic Gardens Seedbank. Seedbank accession records can be found at <https://doi.org/10.26197/5dc21fa4f3b62>

| **Supplementary Table 2.** PCR parameters for amplification of nuclear ribosomal DNA internal transcribed spacer (ITS) regions used to produce *Plantago* phylogenetic tree. | |
| --- | --- |
| **Primers** |  |
| Forward ITS Primer (5’→ 3’) | ACGAATTCATGGTCCGGTGAAGTGTTCG |
| Reverse ITS Primer (5’→ 3’) | TAGAATTCCCCGGTTCGCTCGCCGTTAC |
|  |  |
| **PCR Conditions** |  |
| Activation | 95 °C for 2 min |
| Amplification Cycles | 24 Cycles |
| Denaturation | 95 °C for 30 sec |
| Annealing & Extension (Two-step PCR) | 72 °C for 1 min |
| Final Extension | 72 °C for 5 min |

|  |  | Mean,  Non-Native  n = 4 | Mean,  Native  n = 8 | *P* value | Figure |
| --- | --- | --- | --- | --- | --- |
| Mucilage Macromolecular Properties | CWE Mucilage Yield  (% w/w) | 4.49 | 2.37 | 0.205 ns | 3A |
|  | CWE Mucilage Proportion  (%) | 31.33 | 16.65 | 0.217 ns | 3B |
|  | HWE Mucilage Yield  (% w/w) | 4.88 | 5.11 | 0.890 ns | 3A |
|  | HWE Mucilage Proportion  (%) | 30.08 | 33.13 | 0.640 ns | 3B |
|  | IAE Mucilage Yield  (% w/w) | 6.23 | 6.97 | 0.732 ns | 3A |
|  | IAE Mucilage Proportion  (%) | 38.58 | 50.22 | 0.225 ns | 3B |
|  | Total Mucilage Yield  (% w/w) | 15.60 | 14.45 | 0.777 ns | 3A |
|  | Water Absorption Capacity (mg/mg) | 8.94 | 10.09 | 0.757 ns | 3C |
| Mucilage Composition | CWE Mucilage  Ara:Xyl | 0.180 | 0.346 | 0.349 ns | 4B |
|  | HWE Mucilage  Ara:Xyl | 0.284 | 0.261 | 0.852 ns | 4B |
|  | IAE Mucilage  Ara:Xyl | 0.362 | 0.496 | 0.348 ns | 4B |
|  | CWE Mucilage  Rha+GalA:Xyl+Ara | 0.581 | 0.833 | 0.477 ns | 4C |
|  | HWE Mucilage  Rha+GalA:Xyl+Ara | 0.147 | 0.196 | 0.366 ns | 4C |
|  | IAE Mucilage  Rha+GalA:Xyl+Ara | 0.058 | 0.175 | 0.180 ns | 4C |
| Endosperm Composition | Mannose Content  (% w/w) | 14.71 | 14.60 | 0.961 ns | 5A |
| Soluble Sugar Content | Soluble Sugar Content  (% w/w) | 3.198 | 3.174 | 0.971 ns | 6B |
| Protein Content | Protein Content  (% w/w) | 14.60 | 24.49 | 0.020 * | Table 1 |
| Lipid Content | Fat Content  (% w/w) | 10.50 | 10.68 | 0.931 ns | Table 1 |
|  | Saturated FA Content  (%) | 17.70 | 16.48 | 0.556 ns | Table 1 |
|  | Unsaturated FA Content  (%) | 82.20 | 83.45 | 0.548 ns | Table 1 |
|  | SFA:UFA | 0.217 | 0.199 | 0.561 ns | Table 1 |
|  | Omega-3 FA Content  (%) | 12.55 | 38.62 | 0.008 ** | Table 1 |
|  | Omega-6 FA Content  (%) | 43.58 | 21.90 | 0.001 ** | Table 1 |
|  | Omega-9 FA Content  (%) | 24.84 | 21.60 | 0.565 ns | Table 1 |
|  | Omega-3:Omega-6 | 0.300 | 2.248 | 0.004 ** | Table 1 |
|  | Alpha-Linolenic Acid Content (% w/w) | 1.59 | 4.27 | 0.045 * | Table 1 |

**Supplementary Table 3.** Outcomes of unequal variance independent *t-*tests comparing key factors tested between Non-Native and Australian Native *Plantago* species. CWE = cold water extractable; HWE = hot water extractable; IAE = intense agitation extractable; FA = fatty acid; ns = not significant; * = P < 0.05; ** = P < 0.01.

**Supplementary Figure 1.** Thickness of anticlinal *Plantago* endosperm cell walls. **A.** Values between x-axis tick marks and violin plots denote the median cell wall thickness measured in that sample. **B.** Dashed horizontal line denotes the grand median cell wall thickness from pooled measurements of each species. Cell wall thickness (n = 15–20) was measured using the measurement package of Adobe Photoshop CC 19.0

**
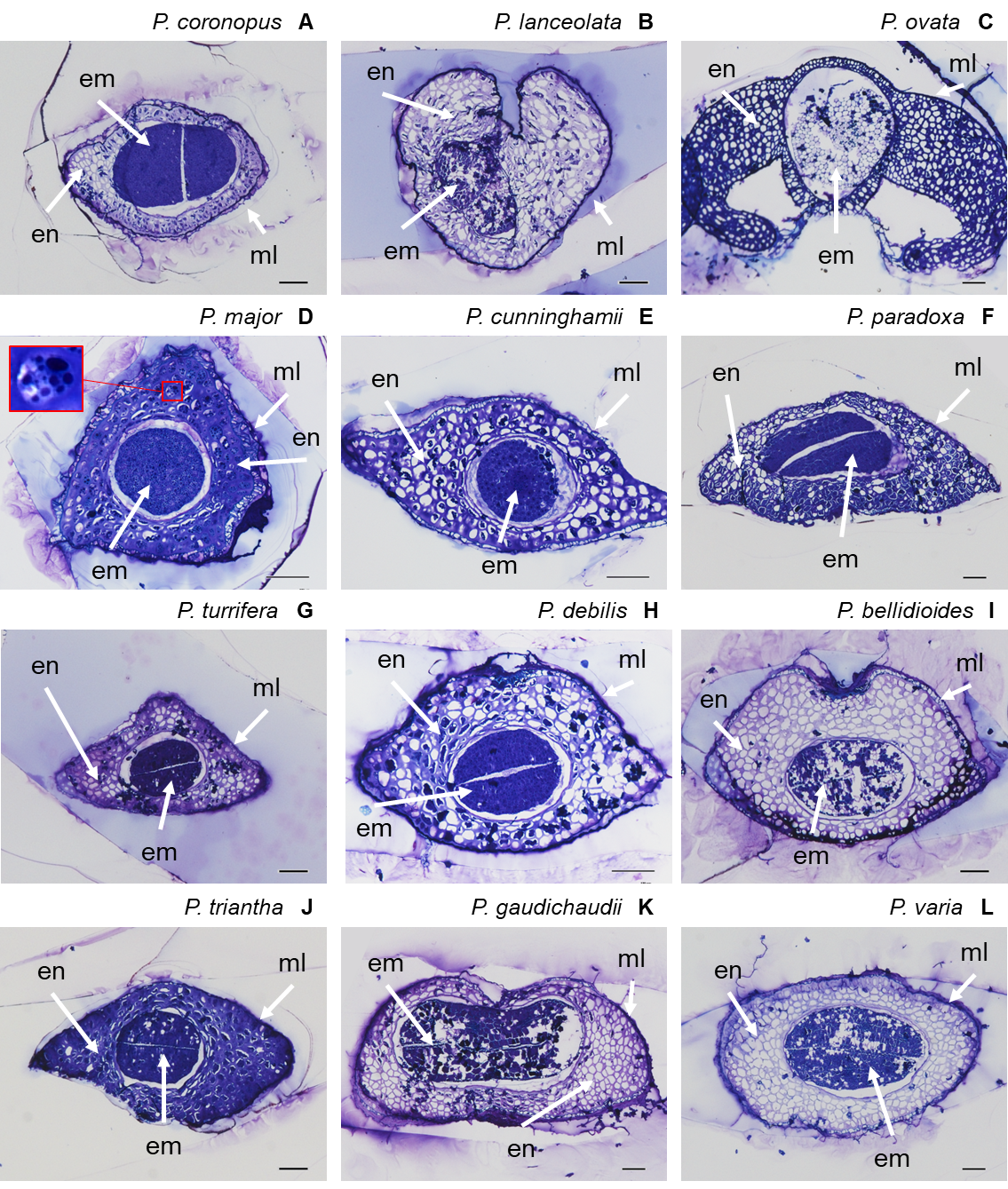
**

**Supplementary Figure 2.** Thin sections of mature *Plantago* seeds stained with Toluidine Blue O to show the major tissue types. Inset in D shows the spherical bodies typically contained within *Plantago* endosperm cells. These spherical bodies are present within endosperm cells of all species but are difficult to retain *in situ* when sectioning, positioning on slide and staining and are thus are sporadically present in images. Scale = 100 μm. Abbreviations: en = endosperm; em = embryo; ml = mucilage layer.

**Supplementary Figure 3.** Heatmap displaying the average relative peak area of soluble sugars extracted from *Plantago* seeds. Retention times of each peak are listed along with their assigned peak name. Values within each cell are the relative peak area of that component in that species.

**Supplementary Figure 4.** Seeds of the *Plantago* species studied here can be grouped as small- or large-seeded, morphometrically and by mass. Seed size is not determined by geographic origin as both size groups contain both native (open circles) and naturalised species (filled circles). Error bars denote one standard deviation.

Abbreviations: Pbel = *P. bellidioides;* Pcor = *P. coronopus;* Pcun = *P. cunninghamii*; Pdeb = *P. debilis*; Pgau = *P. gaudichaudii;* Plan = *P. lanceolata;* Ppar *= P. paradoxa;* Ptri = *P. triantha;* Ptur *= P. turrifera;* Pvar = *P. varia.*


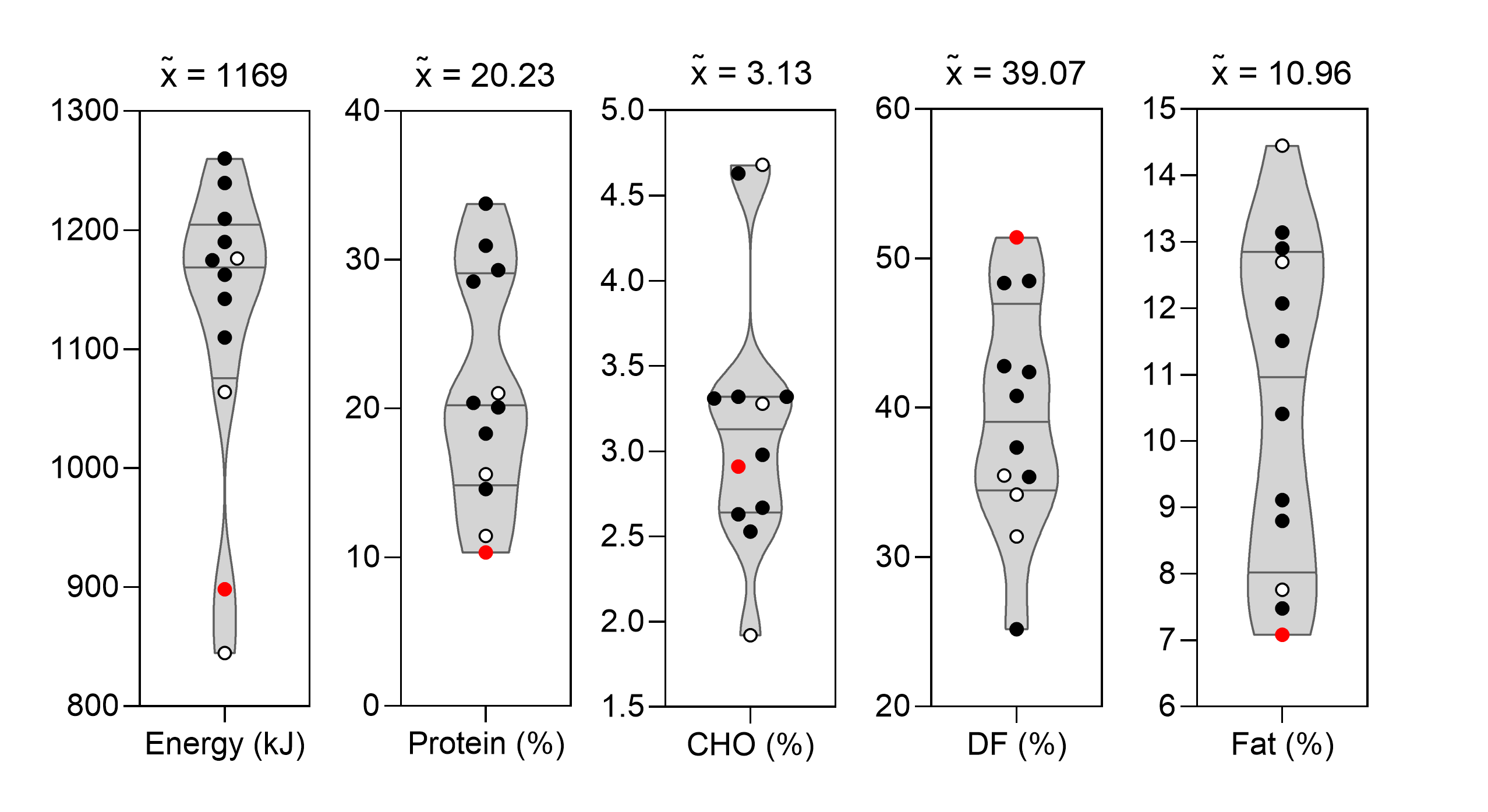


**Supplementary Figure 5.** Truncated violin plots displaying the data range of nutrient content in seeds of the 12 *Plantago* species studied here. Native species are denoted by black points, naturalised species are white points and the commercial species, *Plantago* *ovata,* is a red point. Median values (x̃) are included above each plot. Abbreviations: CHO = carbohydrates; DF = dietary fibre.


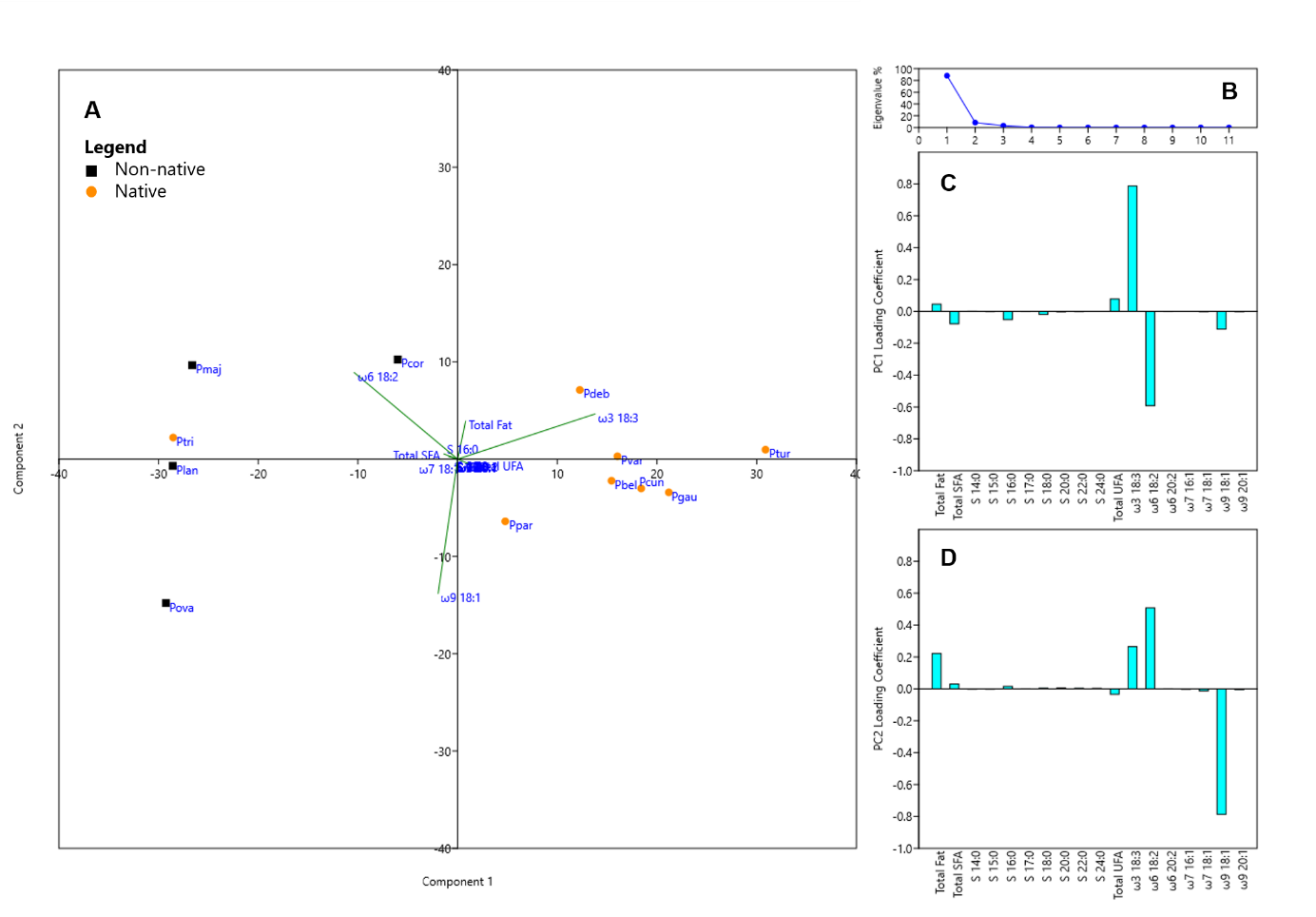


**Supplementary Figure 6.** Principle component analysis of fatty acid (FA) profiling. **A.** PCA biplot of PC1 and PC2 showing FA factors leading to species separation. **B.** Scree plot showing that PC1 and PC2 account for 98% of variance observed. **C.** Loading plot of PC1 and **D.** PC2. In PC1, most separation is due to a relationship between omega-3 (ω3 18:3) to omega-6 FAs (ω6 18:2) (89% of variance) while in PC2 the relationship is between omega-6 (ω6 18:2) and omega-9 FAs (ω9 18:1) (8% of variance).

Abbreviations: Pbel = *P. bellidioides;* Pcor = *P. coronopus;* Pcun = *P. cunninghamii*; Pdeb = *P. debilis*; Pgau = *P. gaudichaudii;* Plan = *P. lanceolata;* Ppar *= P. paradoxa;* Ptri = *P. triantha;* Ptur *= P. turrifera;* Pvar = *P. varia;*SFA = saturated fatty acids; UFA = unsaturated fatty acids.

**
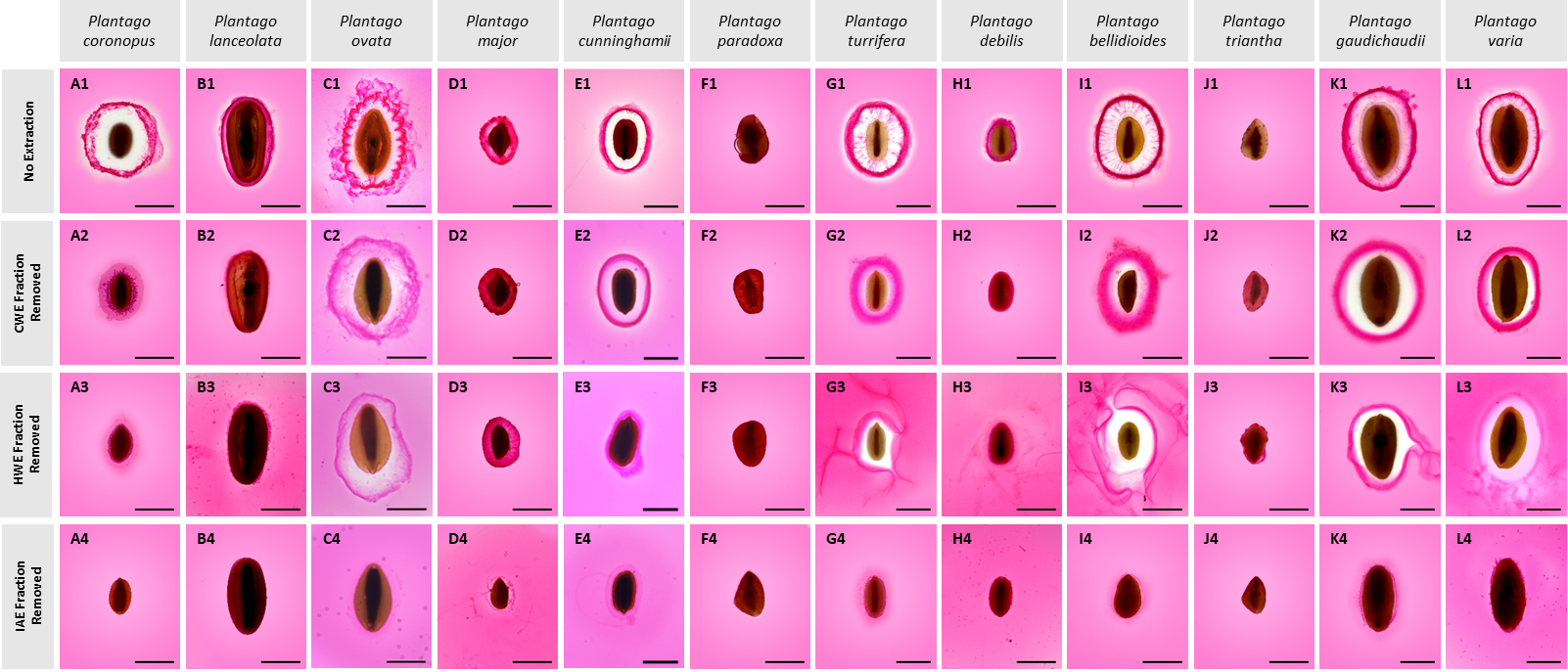
**

**Supplementary Figure 7.** Appearance of the mucilage envelope of each *Plantago* species after each step of fractionation protocol and staining with ruthenium red. Scale = 1 mm. CWE = cold water extractable; HWE = hot water extractable; IAE = intense agitation extractable.

**
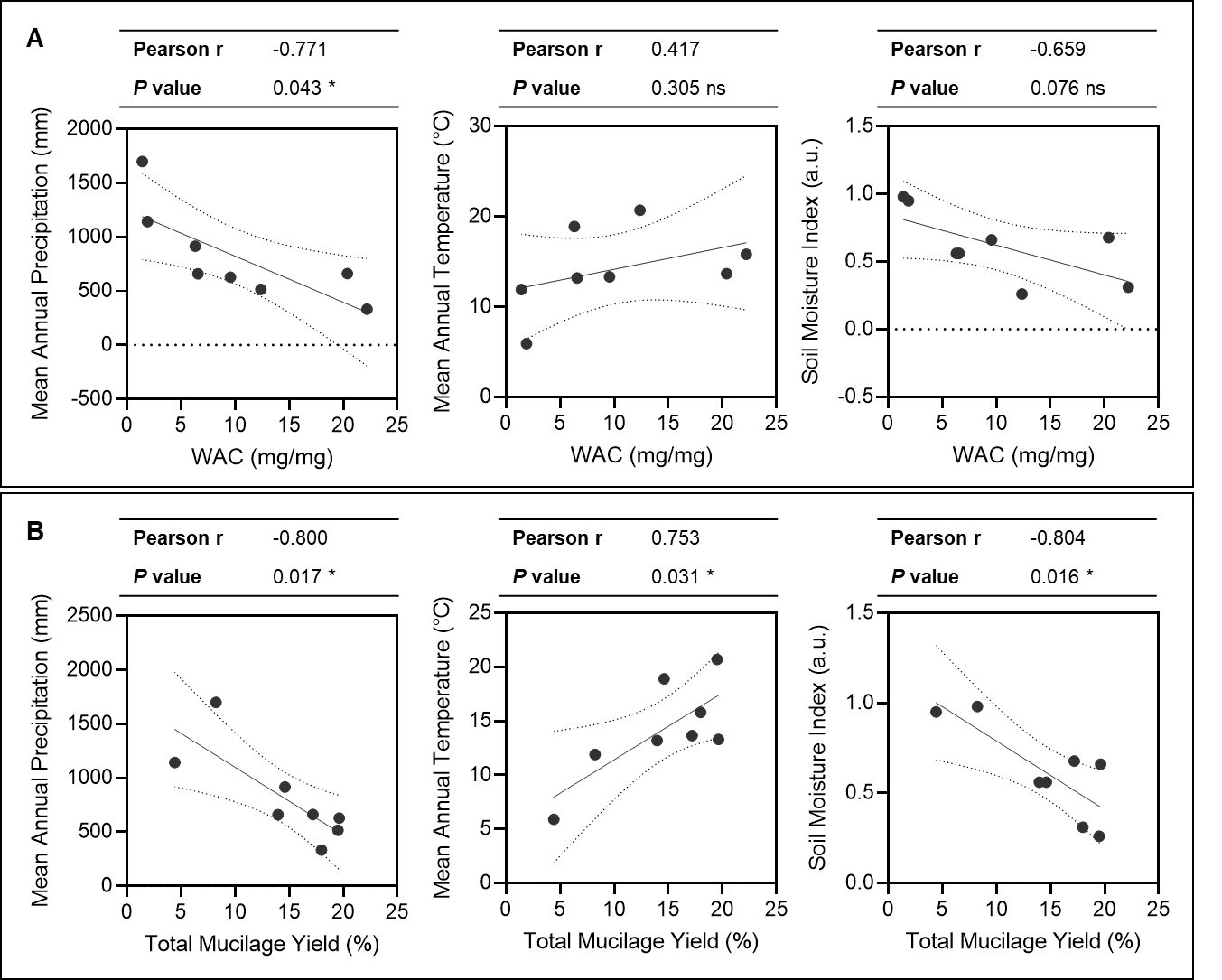
**

**Supplementary Figure 8**. Correlation studies between water absorption capacity (WAC) and total mucilage content of Australian native *Plantago* species and key environmental factors. Solid line is the linear regression, while dotted lines are the 95% confidence interval. Included above each plot is the Pearson r correlation value and the *P* value of the correlation.

Mean annual precipitation, mean annual temperature and soil moisture index data were obtained in May 2021 from the Atlas of Living Australia (ALA) database at <http://www.ala.org.au> for each of the collection locations.

As their exact origins (and associated environmental factors) are unknown, naturalised and commercial species are not included in the correlation study.
